# Gesture-controlled reconfigurable metasurface system based on surface electromyography for real-time electromagnetic wave manipulation

**DOI:** 10.1515/nanoph-2024-0572

**Published:** 2025-01-06

**Authors:** Junzai Chen, Weiran Li, Kailuo Gong, Xiaojie Lu, Mei Song Tong, Xiaoyi Wang, Guo-Min Yang

**Affiliations:** College of Electronic and Information Engineering, Tongji University, Shanghai 200092, China; Key Laboratory for Information Science of Electromagnetic Waves, School of Information Science and Technology, Fudan University, Shanghai 200433, China; College of Electronic and Information Engineering, Shanghai Institute of Intelligent Science and Technology, Tongji University, Shanghai 200092, China

**Keywords:** metasurface, surface electromyography, convolutional neural network, gesture recognition

## Abstract

Gesture recognition plays a significant role in human-machine interaction (HMI) system. This paper proposes a gesture-controlled reconfigurable metasurface system based on surface electromyography (sEMG) for real-time beam deflection and polarization conversion. By recognizing the sEMG signals of user gestures through a pre-trained convolutional neural network (CNN) model, the system dynamically modulates the metasurface, enabling precise control of the deflection direction and polarization state of electromagnetic waves. Experimental results demonstrate that the proposed system achieves high-precision electromagnetic wave manipulation, in response to different gestures. This system has significant potential applications in intelligent device control, virtual reality systems, and wireless communication technology, and is expected to contribute to the advancement and innovation of HMI technology by integration of more advanced metasurfaces and sEMG processing technologies.

## 1 Introduction

In recent years, the field of human-machine interaction (HMI) has undergone significant advancements, driven by the integration of cutting-edge technologies such as machine learning, computer vision, and advanced sensor systems [1], [2], [3], [4]. One of the most promising areas within this domain is the development of gesture control systems, which allow users to interact with devices and environments through intuitive hand movements. This technology has found widespread applications in various sectors, including virtual reality, smart homes, and healthcare, significantly enhancing user experience and system efficiency [5], [6], [7], [8], [9].

Metasurfaces, as an emerging class of artificial materials, have garnered considerable attention due to their natural benefits of low cost, low profile and the strong capability to manipulate electromagnetic waves in unprecedented ways [10], [11], [12], [13]. By engineering the geometric properties of subwavelength structures on a surface, metasurfaces can achieve functionalities that surpass those of conventional materials, such as anomalous reflection [14], [15], absorption [16], [17], polarization conversion [18], [19], [20], [21], radar cross section (RCS) reduction [22], [23], near-field focusing and spectrum shifting [24], [25]. These unique properties have led to a surge of interest in leveraging metasurfaces for applications ranging from wireless communication systems [26], [27], [28], [29], [30], radar sensing systems to optical systems [31], [32], [33], [34], [35].

The integration of gesture control with metasurfaces presents an exciting opportunity to create intelligent metasurfaces platforms that can dynamically respond to user inputs [36], [37]. Gesture controlled metasurfaces have the potential to revolutionize various industries by enabling users to manipulate the properties of electromagnetic waves in real-time through simple hand gestures. For instance, in the communication systems, gesture control could allow for dynamic reconfiguration of antenna beams, enhancing signal strength and directionality based on user needs. Similarly, in the field of virtual and augmented reality, gesture-controlled metasurfaces could provide users with more intuitive and immersive interaction experiences.

Many gesture recognition methods have been studied in various platforms, such as computer vision methods [38], [39], [40], [41], [42], radio frequency methods [43], [44], [45], wearable device methods [46], [47], [48], [49]. Computer vision methods use a camera to capture gesture images or video and perform gesture recognition through image processing and deep learning algorithms. Radio frequency methods use radio waves (e.g., radar or Wi-Fi) to capture hand or body movements and recognize gestures by analyzing changes in reflected signals. And wearable device methods use sensors worn on the user’s body to capture hand or body movements and physiological signals, and perform gesture recognition through pattern recognition algorithms. However, computer vision methods are affected by environmental factors such as lighting conditions and background complexity, and camera capture of user images can raise privacy concerns. Radio frequency methods have relatively low spatial resolution, which makes it difficult to capture subtle gesture movements, and are susceptible to interference from other wireless devices. Wearable device methods can effectively solve these problems and become an effective method for gesture recognition. The surface electromyography (sEMG) signals, as a special kind of physiological electrical signals, can effectively reflect the behavioral intentions of the human body, and are natural, direct, and non-invasive, providing a reliable source of data for gesture recognition, which is well suited to the field of HMI [50], [51], [52], [53], [54], [55].

**Figure 1:**
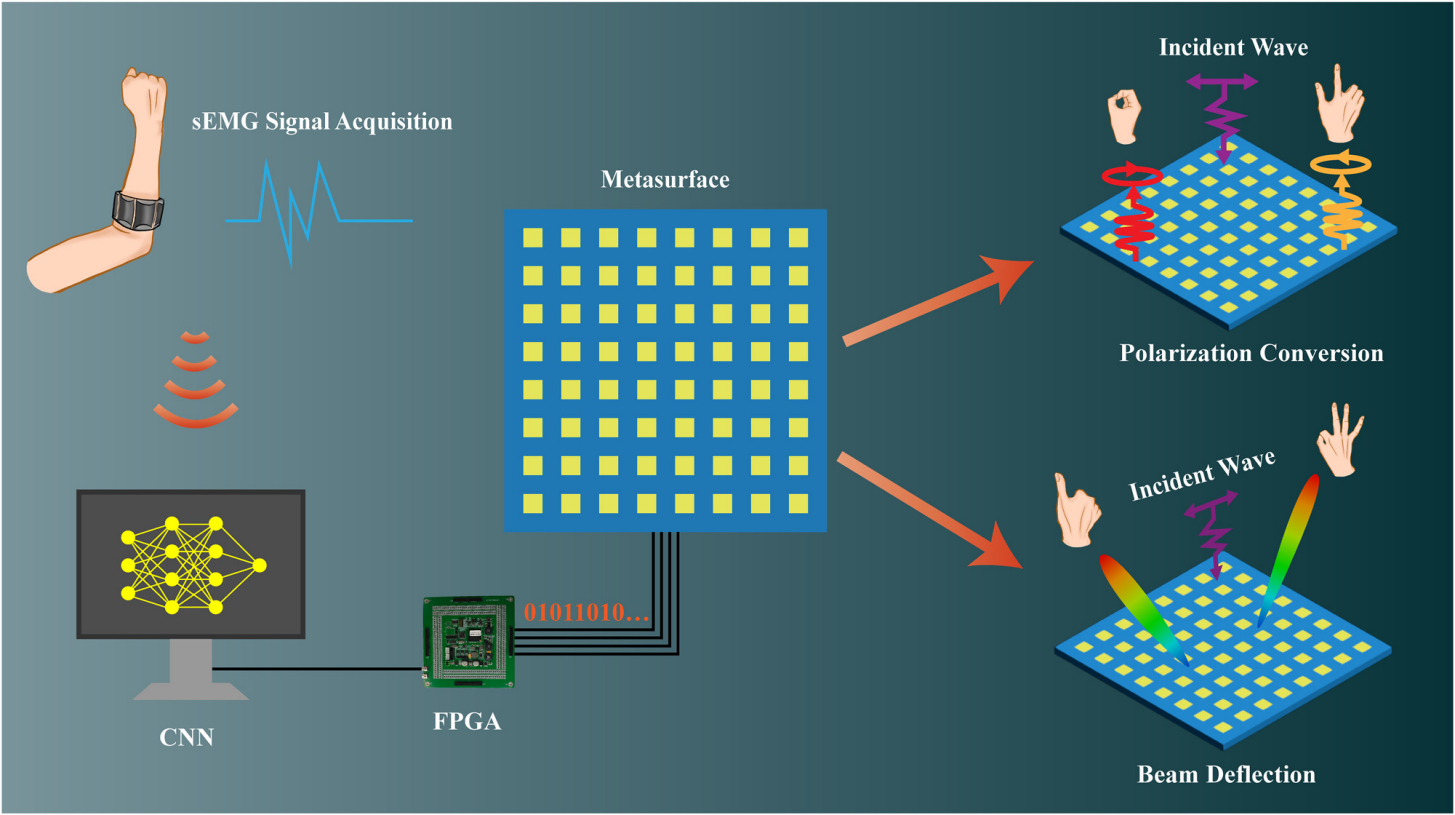
Schematic diagram of the proposed gesture-controlled reconfigurable metasurface system. It comprises a sEMG armband, a computer, an FPGA, and a 2-bit reconfigurable metasurface. By performing different gestures, the metasurface can realize polarization conversion and beam deflection functions dynamically.

In this paper, a gesture-controlled reconfigurable metasurface system based on sEMG for real-time beam deflection and polarization conversion is proposed as shown in Figure 1. By collecting the sEMG signals with an armband and recognizing the sEMG signals of the user’s gestures with a pre-trained convolutional neural network (CNN) model, dynamic modulation of the metasurface to control the deflection direction and polarization state of the electromagnetic waves can be realized. Distinct from conventional CNN architectures, this paper innovatively incorporates batch normalization layers, rectified linear unit (ReLU) activation layers, and dropout layers, along with a piecewise learning rate strategy. These enhancements significantly improve the model performance in eliminating the tedious process of manual feature extraction and reducing the risk of overlooking valuable information in the signals, which results in high classification accuracy, strong robustness against windowing errors and signal strength variations. In the metasurface design, an innovative 2-bit reconfigurable metasurface using only 3 PIN diodes is proposed to balance the design complexity and phase quantization error. Additionally, visualized wireless communication experiments to transmit an image with QPSK modulation to receivers at different positions or receivers of different polarized antennas are designed to demonstrate the proposed real-time system. The comprehensive exploration of this new system of controlling electromagnetic waves by gesture sEMG signals harnesses the unique properties of metasurfaces and the intuitiveness of gesture control, which creates innovative systems that push the boundaries in terms of user interaction and electromagnetic wave manipulation, highlighting their potentials as next-generation material platforms for HMI systems. Furthermore, the system represents a pioneering integration of cutting-edge technologies from multiple disciplines, including biomedical engineering (sEMG signal acquisition and processing), artificial intelligence (gesture recognition algorithms), and electromagnetics (metasurface design and control), achieving interdisciplinary collaboration and innovation.

## 2 Results

### 2.1 sEMG gesture recognition

The sEMG signals represent the integrated output of superficial muscle electromyography signals and the electrical activity observed on the nerve trunk at the skin surface. This combined signal can, to some extent, reflect neuromuscular activity. In comparison to needle-electrode electromyography, sEMG offers the benefits of being non-invasive, simple to operate in terms of measurement, and requiring minimal effort to perform. The sEMG-based acquisition device measures the electrical signals generated by muscles during activity through electrodes placed on the skin surface, which is a widely used tool in the fields of medicine, rehabilitation, sports science, and HMI.

The sEMG PRO armband, developed by Sichiray Technology Co., Ltd., is utilized for the acquisition of sEMG signals in this work. The armband is designed to be worn on the forearm and comprises eight channels to collect, amplify and filter the superficial muscle electromyography signals. Meanwhile, the armband is also equipped with software to extract sEMG data after windowing segmentation from the device directly. Typical original sEMG signal amplitude is in the range of 0–5,000 uV, and the sEMG PRO armband used in this work has an output voltage of 0–3.3 V, after an electrode amplification of around 2,000 times, a biasing voltage, and an internal ADC with 12-bit resolution. Due to the presence of a bias voltage, when the arm is relaxed, the output value remains around a DC value with random noise.

**Figure 2:**
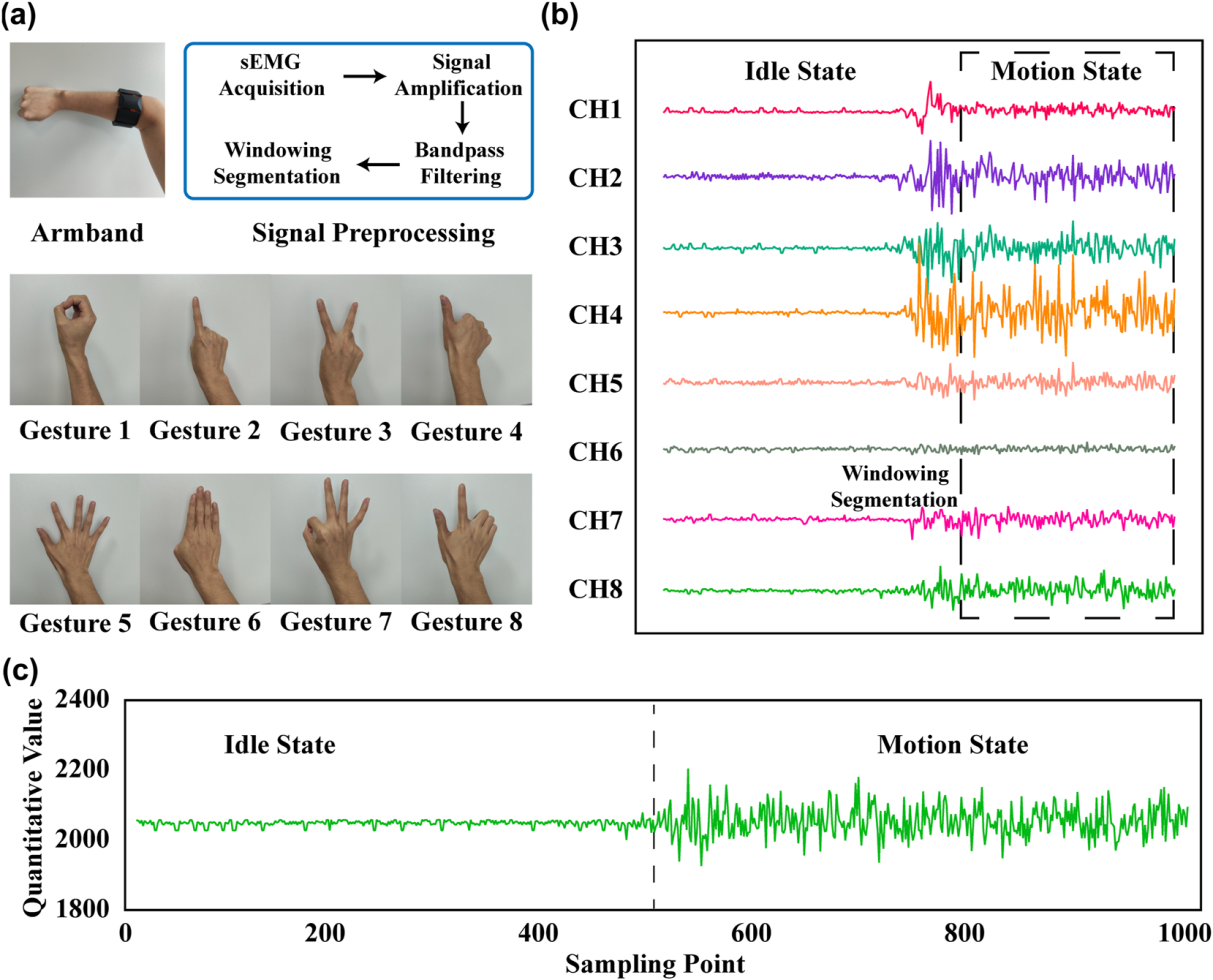
sEMG signals acquisition and processing. (a) The position of the armband worn, signal processing process of the armband worn and 8 predefined gestures to be recognized. (b) Waveforms and windowing segmentation of the eight-channel of sEMG signals of gesture 1. (c) The detailed specification of the sEMG.

Figure 2a illustrates the position of the armband worn and the signal processing flow when the sEMG PRO armband is employed to collect the sEMG data. Additionally, it depicts eight gestures, defined as gestures 1–8, which are selected for real-time manipulation of metasurface functions in this paper. Figure 2b gives the waveforms and windowing segmentation of the eight channels of sEMG signals acquired when performing gesture 1. Varying signal intensities across different channels are primarily due to differences in muscle activity levels at various locations on the arm. We use a single-channel example to illustrate the differences in the acquired signal at varying muscle activity levels, namely, idle state (no motion) and motion state, as shown in Figure 2c. By carefully processing sEMG signals and extract their features, the hand gesture can be recognized.

In the field of gesture recognition, traditional machine learning methods often rely on complex manual feature extraction processes. These processes are not only time- consuming and labor-intensive, but also difficult to comprehensively capture the complex features present in sEMG signals. As a type of bioelectrical signal, the morphology and frequency characteristics of sEMG signals vary considerably depending on the specific muscle activity involved. These variations contain a wealth of information that can be utilized to recognize a range of gestures. Deep learning model, particularly CNN, is capable of automatically extracting features from raw data through multilayer neural networks, thereby reducing the reliance on manual feature design. Concurrently, CNN demonstrates efficacy in processing high-dimensional data and complex pattern recognition tasks. Consequently, CNN has emerged as a highly effective tool in the field of gesture recognition, demonstrating superior performance in terms of automation, accuracy, and the handling of complex data compared to traditional methods.

**Figure 3:**
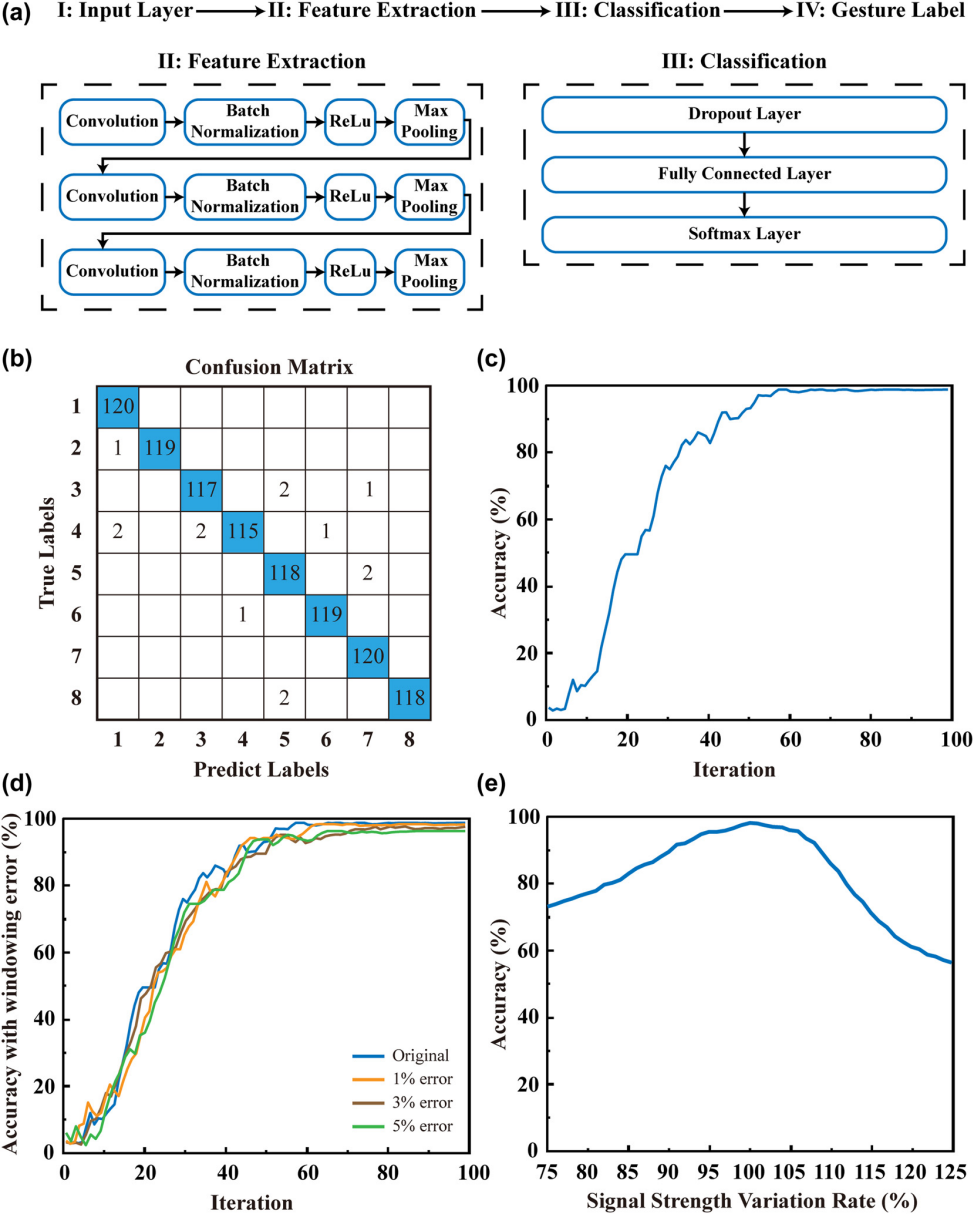
Construction and training results of the CNN. (a) CNN structure of recognition model. (b) The confusion matrix of model. (c) The accuracy of model. (d) Model accuracy with different windowing errors. (e) Model accuracy under varying signal strengths.

The CNN architecture utilized in this work is illustrated in Figure 3a. The CNN model comprises an input layer, three groups of convolutional layers, batch normalization layers, ReLu activation layers and pooling layers for feature extraction. It also includes a dropout layer, a fully connected layer, and a softmax layer for gesture classification. The input layer receives the sEMG signal data, and the convolutional layer extracts local features from the input data by applying multiple convolutional kernels. The inclusion of batch normalization layers and ReLu activation layers serve to accelerate the training process and introduce nonlinearities. The maximum pooling layer performs downsampling to reduce the number of features and retain those that are most pertinent. The dropout layer randomly discards some neurons with a probability of 0.5, thereby reducing the risk of overfitting. The next component of the network is a fully connected layer with an output layer size of eight, representing the eight distinct gesture categories. Subsequently, the output is transformed into a probability distribution through the application of a Softmax layer. The entire network is trained using the Adam optimizer with an initial learning rate of 0.001, and the model performance is further optimized through the implementation of a segmented learning rate tuning strategy.

The sEMG signal data for the eight gestures is collected using a sEMG PRO armband. Each gesture is performed for a duration of 5 s, followed by a three-second relaxation period. Each gesture is repeated 24 times. The dataset comprises 960 samples, 120 for each gesture, obtained through the software provided with the sEMG PRO armband. Subsequently, the dataset is employed for the training and evaluation of the proposed CNN model. Figure 3b and c illustrate the confusion matrix and the accuracy of the trained model, respectively, showing that the proposed CNN model can achieve 98.54 % accuracy after 100 iterations.

The sEMG signals typically precede limb movements by 30–150 ms. During sEMG data acquisition, we ensure that recording begins only after the gesture has been executed to maximize the inclusion of valid data within the window and minimize windowing error. If a windowing error presents by accident, resulting in the capture of invalid data, such occurrences are expected to represent only a very small fraction of the total dataset. To simulate the effect of windowing error, we intentionally introduced invalid signals by replacing the first 1 %, 3 %, and 5 % of the samples for each gesture in the dataset with invalid data, effectively mimicking potential windowing errors. The model’s training accuracy, as shown in Figure 3d, decreased by 0.48 %, 1.32 %, and 2.58 %, respectively, compared to the original accuracy, which demonstrates the proposed algorithm exhibiting strong robustness against windowing error. The detecting error for different signal strengths is also studied by artificially amplifying or reducing the signal strengths and subsequently input the signal into the recognition model, and the results are shown in Figure 3e. When the variation of signal strengths does not exceed plus or minus 10 %, the recognition accuracy is still higher than 90 %, which demonstrates that the proposed algorithm also exhibits strong robustness against signal strengths variations.

### 2.2 Design of the 2-bit reconfigurable metasurface

**Figure 4:**
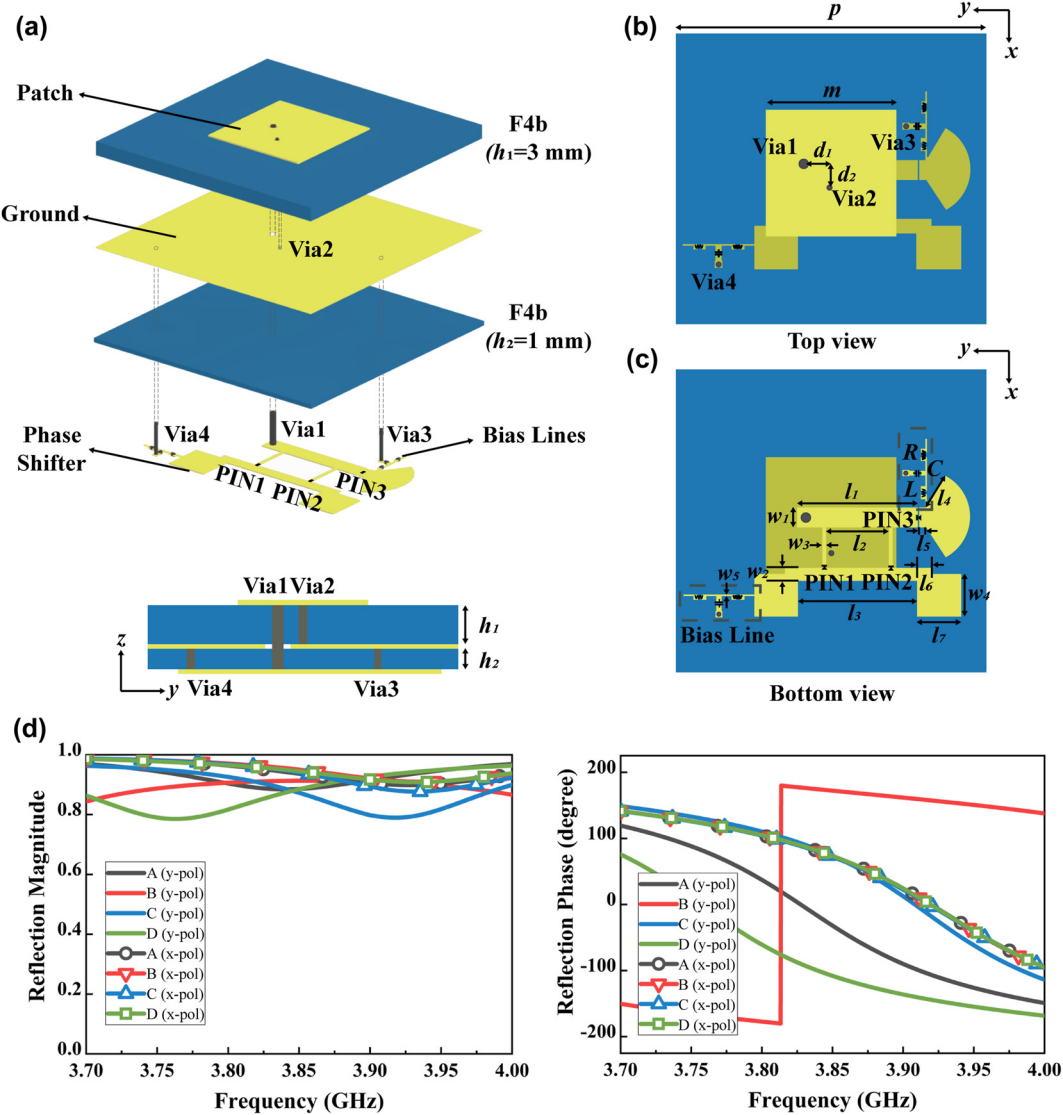
Design of 2-bit reconfigurable metasurface unit cell. (a) Exploded view of the metasurface unit cell. (b) Top view of metasurface unit cell. (c) Bottom view of metasurface unit cell. (d) Simulated reflection magnitude and phase responses of the unit cell for both *x*-polarized and *y*-polarized incidences.

The designed metasurface unit cell and its simulated performance are shown in Figure 4. Figure 4a depicts the three-dimensional structure of the 2-bit reconfigurable metasurface cell, which consists of three layers of metal, two layers of substrate and four metallic vias. These vias are designed to facilitate the connection between the three metal layers. Both substrates are composed of F4b material (*ɛ*_*r*_ = 2.47, tan *δ* = 0.002), with a thickness of 3 mm and 1 mm, respectively. The top surface features a square metal patch, as illustrated in Figure 4b, which is used to receive the incident wave and rescatter it into freespace. The bottom layer is a 2-bit reflective phase shifter, with two bias lines for modifying the reflective phase state, as illustrated in Figure 4c. Three diodes (MA4AGFCP910), designated as PIN 1, PIN 2, and PIN 3, have been integrated on the phase shifter. Two bias lines are used to independently control the three diodes, and the RLC lumped element design blocks radio frequency signal and allows direct circuit signal to pass through, with *R* = 47 Ω, *L* = 68 pF, and *C* = 33 nH. The middle metal layer serves the function of a common grounding layer for top and bottom layers. By dividing the unit structure into a radiation part and a phase shift part, the PIN diodes can be placed on the bottom layer of the metasurface to minimize the unnecessary scattering effects, enhance system reliability, and simplify the design process.

The unit cell is modeled and simulated using simulation software HFSS. To realize a 2-bit reflection phase shift, four distinct coding states, designated as “A”, “B”, “C” and “D”, are defined. Table 1 illustrates the correspondences between the operational states of the diodes and the four encoding states. Figure 4d depicts the simulated amplitude and phase response of the unit cell. The results demonstrate that the reflection loss of the y-polarized incident wave is less than 1.5 dB within working frequency range of 3.85–3.90 GHz. Additionally, we are able to find the phase difference of adjacent states is approximately 90°. In the case of x-polarized incident wave, the reflection loss is less than 0.6 dB, and the reflection phases of the four states are roughly equal.

**Table 1:** Correspondence between states and PIN diodes.

State	A	B	C	D
PIN1, PIN2	OFF	OFF	ON	ON
PIN3	OFF	ON	OFF	ON

In accordance with the metasurface unit cell design outlined above, a metasurface array is constructed consisting of 8 × 8 elements. Each metasurface element is controlled by two bias circuits that can switch between the four coding states of “A”, “B”, “C” and “D” to achieve beam deflection and polarization conversion functions.

**Figure 5:**
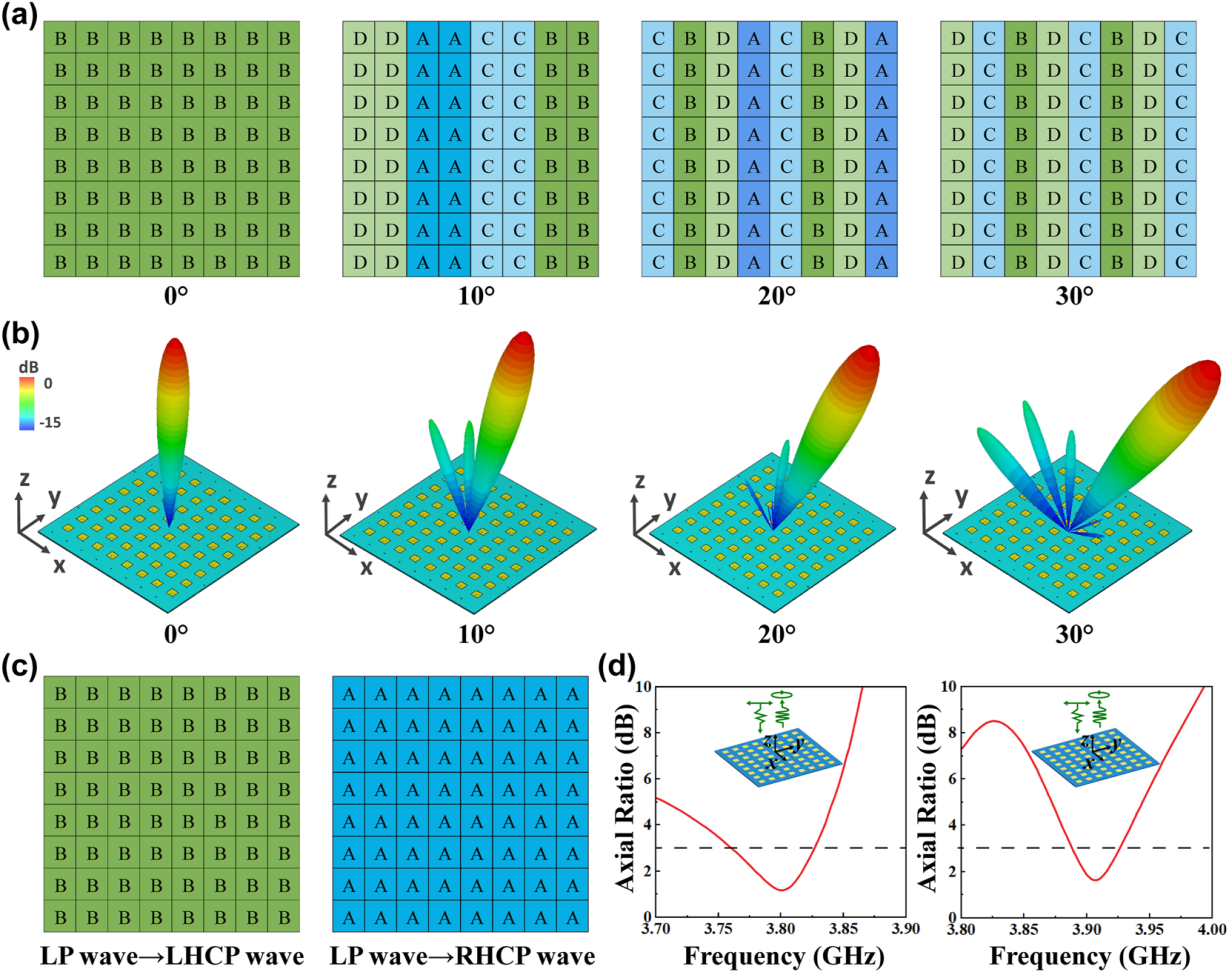
Coding matrices and simulation results of beam deflection with *y*-polarized incidence and polarization conversion with 45° LP incidence wave. (a) Coding matrices of the metasurface at varying deflection angles: 0°, 10°, 20° and 30°. (b) Far-field patterns simulation results for 0°, 10°, 20° and 30°. (c) Coding matrices for converting 45° LP wave to LHCP and RHCP wave. (d) Simulated results of AR for the reflected LHCP and RHCP wave.

Figure 5 illustrates the full-wave simulated beam deflection and polarization conversion function results. Figure 5a and b illustrate the coding matrices of the metasurface for different deflection angles, accompanied by the corresponding far-field simulation patterns. The four different phase gradients (0°, 90°, 180°, and 270°) provided by the coding matrices result in gradually deflected scattering patterns, which are shifted from 0° to 30° in approximately 10° steps at 3.80 GHz, with working frequency ranges from 3.73 to 3.83 GHz. By adjusting among the “A”, “B”, “C”, and “D” states, we can realize reconfigurable beam deflection effectively. Figure 5c illustrates the coding matrices for the polarization conversion function, while Figure 5d shows the simulation results. According to the phase characteristics in Figure 4d, it can be observed that the *y*-direction phase response is approximately 90° in advance of that in the *x*-direction when all metasurface cells are set to the “B” coding state. Consequently, the linearly polarized (LP) incident wave is converted into left-handed circularly polarized (LHCP) wave, and the axial ratio (AR) is less than 3 dB from 3.75 to 3.82 GHz. When all elements are set to the “A” coding state, it is clear that the *y*-direction phase response is delayed by around 90° relative to the *x*-direction. Thus, the LP incident wave is transformed to right-handed circularly polarized (RHCP) wave, and the AR of the “A” arrangement is also less than 3 dB, while the frequency range is 3.88–3.92 GHz.

### 2.3 Experimental verification

**Figure 6:**
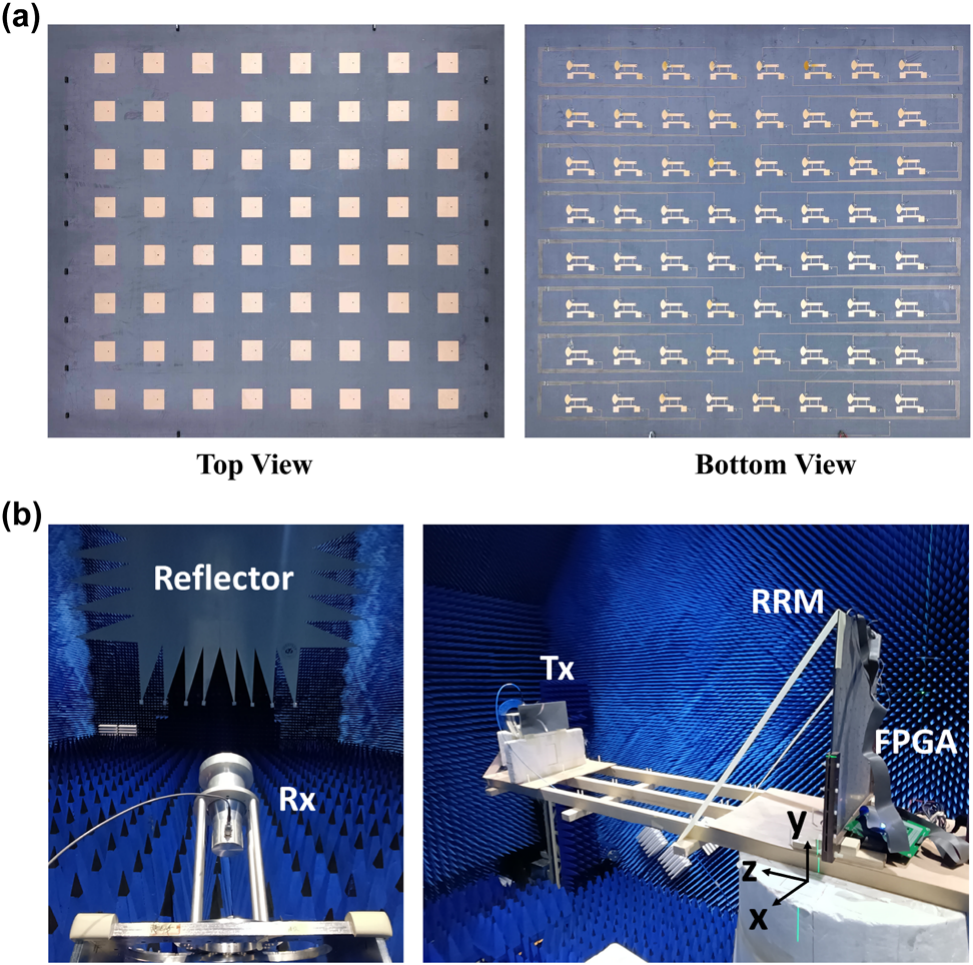
Fabrication of metasurface prototype and experimental setup. (a) Physical photographs of the 2-bit metasurface prototype. (b) Photograph of measuring setup for beam deflection and polarization conversion by gesture control of metasurface.

One able-bodied male subject (age 26) participated in the experiment. Informed consent was obtained prior to the start of the study. Figure 6 shows the fabricated metasurface prototype and experimental set-up. A 2-bit reconfigurable metasurface comprising 8 × 8 cells is fabricated and tested, as illustrated in Figure 6a. The 192 PIN diodes integrated in the metasurface can be individually regulated by applying the bias voltages. The coding matrices for the various functions are pre-stored in the FPGA platform’s memory.

Figure 6b illustrates the experimental configuration of the gesture-controlled reconfigurable metasurface system for beam deflection and polarization conversion functions. The sEMG signals are captured by the sEMG PRO armband and the pre-trained CNN is stored in a laptop computer, respectively. Upon recognition of the captured gesture sEMG signals by the CNN, the laptop computer loads the coding matrix corresponding to the gesture onto the metasurface through the FPGA in order to control the diode states. For reflection coefficient, *T*_*x*_, *ϕ*_*x*_, *T*_*y*_ and *ϕ*_*y*_ are used to represent its amplitude and phase. The AR of the scattered wave can be obtained through the calculation of

(1) $$\text{AR}=\sqrt{\frac{{\left|T_{x}\right|}^{2}+{\left|T_{y}\right|}^{2}+\sqrt{a}}{{\left|T_{x}\right|}^{2}+{\left|T_{y}\right|}^{2}-\sqrt{a}}}$$

(2) $$a={\left|T_{x}\right|}^{4}+{\left|T_{y}\right|}^{4}+2{\left|T_{x}\right|}^{2}{\left|T_{y}\right|}^{2}\cos\left(2\Delta \phi \right)$$

**Figure 7:**
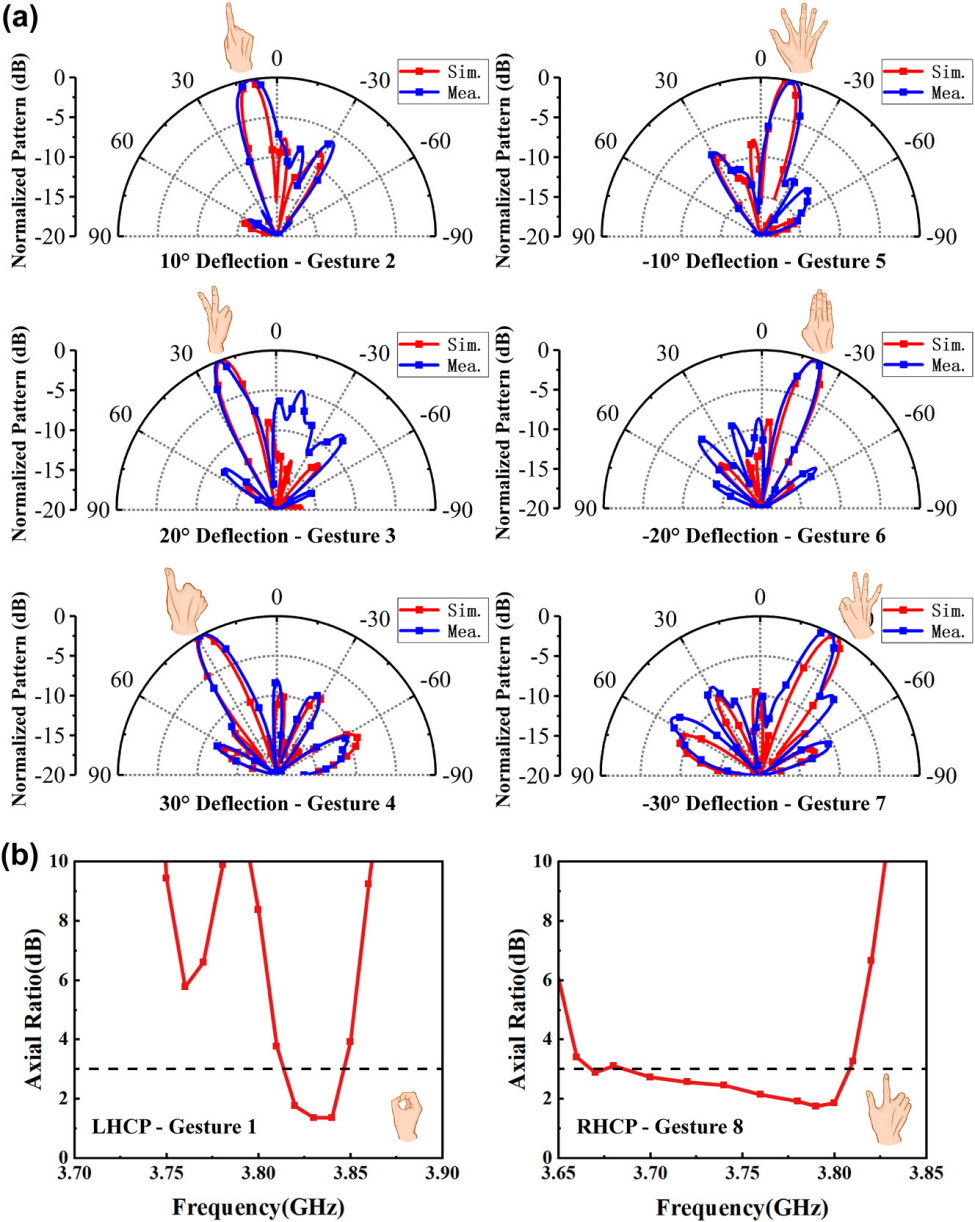
Experimental results. (a) Results of scattering patterns of the gesture-controlled beam deflection function from −30° to 30°. (b) Results of AR of the gesture-controlled polarization conversion function.

Figure 7 plots the measured results for different gestures. Figure 7a illustrates the results of the gesture-controlled beam deflection function. At 3.83 GHz, the deflection angles of −30° to +30° correspond to six different control gestures at 10° intervals. The deviations of the measured direction of the maximum beam amplitude from the simulated results are 3°, 0°, −3°, 0°, −1°, 1°, and −2°, respectively, which are very small. Figure 7b illustrates the results of the gesture-controlled polarization conversion function. Following the recognition of gesture 1, the AR of the LP to LHCP is found to be less than 3 dB from 3.82 to 3.85 GHz. Similarly, following the recognition of gesture 8, the AR of the LP to RHCP conversion function is observed to be less than 3 dB from 3.68 to 3.82 GHz. The minor discrepancies between the measured and simulated results may be attributed to the fabrication errors and the influence of the testing environment. The measurements show that the conversion functions of LP to LHCP and RHCP can be achieved through the switch between gestures 1 and 8.

**Figure 8:**
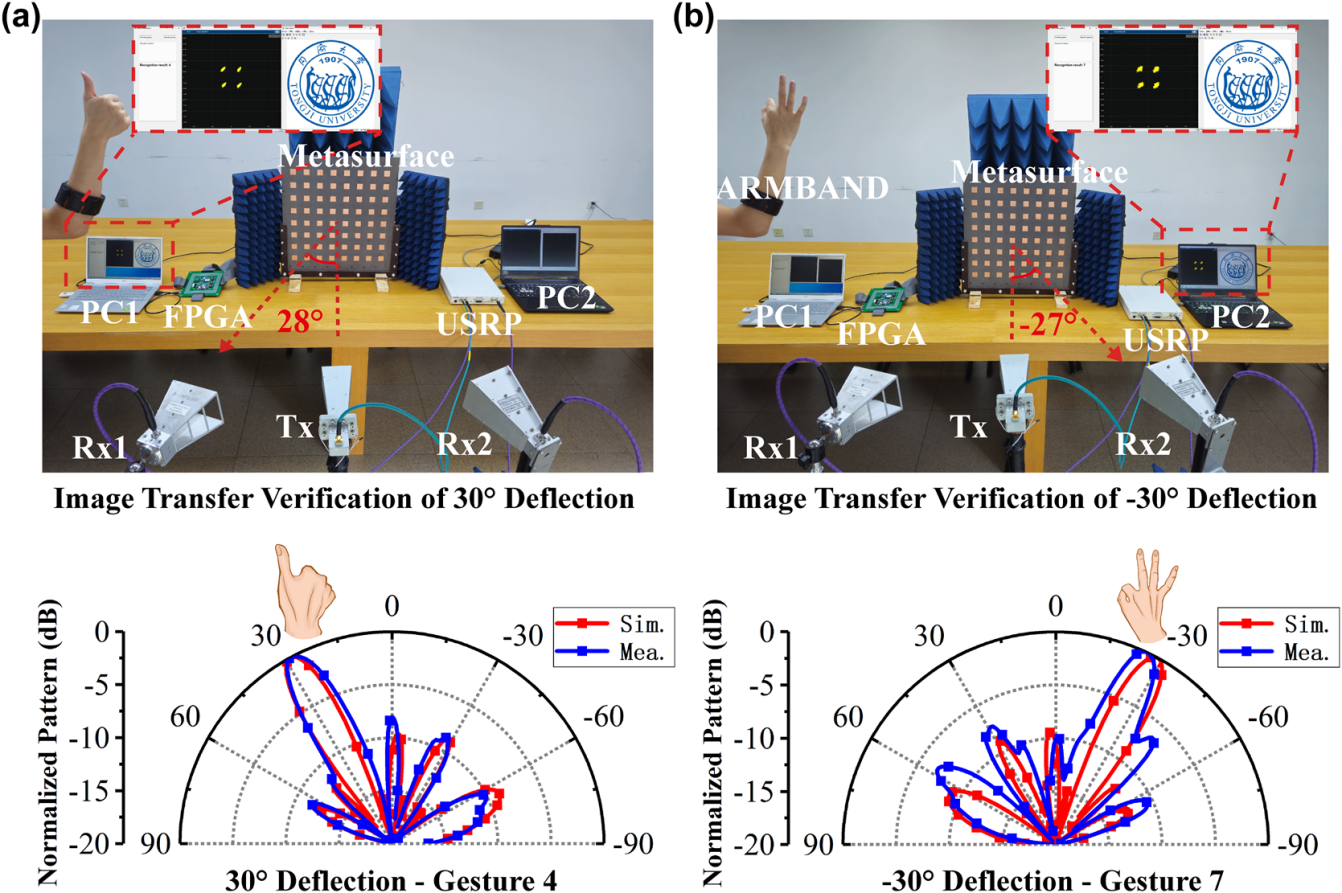
The visualized beam deflection transmission experiment based on the gesture-controlled reconfigurable metasurface system. (a) Transmit the image to receiver at 28°. (b) Transmit the image to receiver at −27°.

**Figure 9:**
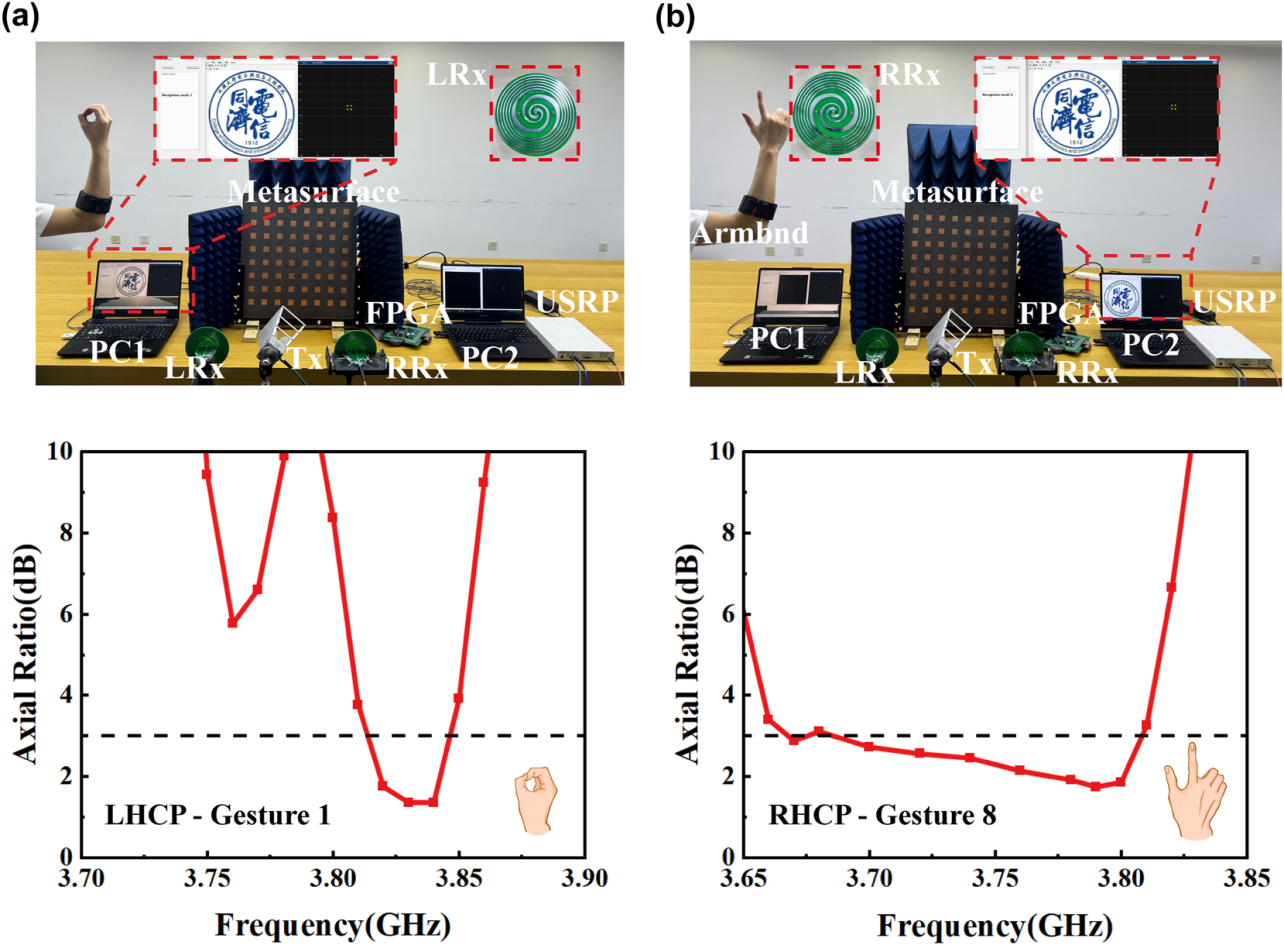
The visualized polarization conversion transmission experiment based on the gesture-controlled reconfigurable metasurface system. (a) LP-LHCP image transmission experiment. (b) LP-RHCP image transmission experiment.

Based on the actual measurements of the metasurface above, a visualized wireless communication experiment to transmit an image to receivers at different locations is designed to investigate the manipulation of beam deflection direction through the use of different gestures. Figure 8a shows that when the gesture 4 is performed, the receiver located at 28° can demodulate the corresponding QPSK signal with a good constellation and display the transmitted image well, while the receiver located at −27° receives a signal with very low power that below the detecting threshold, which demonstrates the scattering beam is scattered to 28° when gesture 4 is present. Similarly, Figure 8b demonstrates the scattering beam is scattered to −27° when gesture 7 is present. A visualized polarization conversion transmission experiment is also designed to demonstrate the gesture-controlled polarization conversion function. The experimental scenario and results are shown in Figure 9, where the LP transmitting antenna is incident at 45° and transmits the images to the LHCP receiving antenna (LRx) and the RHCP receiving antenna (RRx), respectively, in order to investigate the manipulation of the metasurface’s polarization conversion function through the use of different gestures. Figure 9a shows that when gesture 1 is performed, the LRx receives the modulated QPSK signal with a good constellation diagram and displays the demodulated image well. Figure 9b shows that when gesture 8 is recognized, the metasurface converts the incident LP wave into a RHCP wave, which is received by the RRx. The two experimental results vividly demonstrate that the proposed system can manipulate the metasurface to achieve the beam deflection function and polarization conversion function through different gestures.

## 3 Discussion

We propose a gesture-controlled reconfigurable metasurface system based on sEMG to achieve real-time beam deflection and polarization conversion. By using an optimized CNN architecture and an innovatively designed metasurface, the proposed system is able to dynamically modulate electromagnetic waves. The experimental results demonstrate that the system can achieve high-precision electromagnetic wave modulation in response to different gestures. The system has considerable potential for application in a number of fields, including wireless communication, smart home and health monitoring, which is anticipated to contribute to the advancement and innovation of HMI technology.

## 4 Methods

The beam deflection function and polarization conversion function measurements are conducted in a microwave chamber, as depicted in Figure 6. For the beam deflection function measurement, the metasurface is placed on a rotatable platform. The transmitting antenna is fixed at a distance of 1.5 m in front of the metasurface simulating the incidence of a plane wave while the far-field area holds the receiving antenna, both of which are *y*-polarization. Scattering patterns are obtained using a vector network analyzer (Keysight P9375A). The setup for measuring the polarization conversion function is analogous to that used for beam deflection, with the exception that the transmitting antenna has a *φ*_*r*_ = 45° rotation in the *z* direction, and the receiving antenna is oriented along *x* and *y* directions.

Figure 8 illustrates the experimental setup of the visualized wireless communication experiment of beam deflection function. The acquisition and recognition of hand gestures are conducted via the sEMG PRO armband and a pre-trained CNN. An image is converted into a sequence of bits and then QPSK modulation is performed. The modulated image signal is transmitted towards the metasurface via the Universal Software Radio Peripheral (USRP, NI USRP-2943R) and the transmitter antenna. The deflection of the beam is contingent upon the specific gesture employed. Two different antennas, Rx1 and Rx2, are located at 28° and −27° to pick up the signals scattered by the metasurface, respectively. These signals are then sent to USRP for QPSK demodulation and image displaying. Figure 9 illustrates the experimental setup of the visualized wireless communication experiment of polarization conversion function. The experimental setup is the same as the beam deflection function, except that the LP transmitting antenna is incident at 45° and the receiver uses LHCP receiving antenna (LRx) and the RHCP receiving antenna (RRx), respectively.
